# Emigration-related attitudes of the final year medical students in Croatia: a cross-sectional study at the dawn of the EU accession

**DOI:** 10.3325/cmj.2014.55.452

**Published:** 2014-10

**Authors:** Ivana Kolčić, Mihaela Čikeš, Kristina Boban, Jasna Bućan, Robert Likić, Goran Ćurić, Zoran Đogaš, Ozren Polašek

**Affiliations:** 1 Department of Public Health, University of Split, School of Medicine, Split, Croatia; 2 Medical student, University of Split, School of Medicine, Split, Croatia; 3 Department of Internal Medicine, University of Zagreb, School of Medicine, Zagreb, Croatia; 4 Unit of Clinical Pharmacology, Department of Internal Medicine, Clinical Hospital Center Zagreb, Zagreb, Croatia; 5 Laboratory for DNA analysis, J. J. Strossmayer University of Osijek, School of Medicine, Osijek, Croatia; 6 Department of Neuroscience, University of Split, School of Medicine, Split, Croatia

## Abstract

**Aim:**

To investigate the emigration-related attitudes of final year medical students in Croatia at the dawn of the EU accession in 2013.

**Methods:**

All final-year medical students at four Croatian medical schools (Zagreb, Rijeka, Split, and Osijek) were invited to participate in a cross-sectional survey on emigration attitudes.

**Results:**

Among 260 respondents (response rate 61%), 90 students (35%) reported readiness for permanent emigration, expecting better quality of life (N = 22, 31%), better health care organization (N = 17, 24%), more professional challenges (N = 10, 14%), or simply to get a job (N = 8, 11%), while the least common expectation were greater earnings (N = 7, 10%). The most common target countries were Germany (N = 36, 40%), USA and Canada (N = 15, 17%), and UK (N = 10, 11%). In a multivariate analysis, readiness for permanent emigration was associated with an interest in undertaking a temporary training abroad (odds ratio [OR] 6.87; 95% confidence interval [CI] 2.83-16.72), while the belief that the preferred specialty could be obtained in Croatia appeared protective against emigration (OR 0.26; 95% CI 0.12-0.59).

**Conclusion:**

Despite shortages of health care workers in Croatia, the percentage of students with emigration propensity was rather high. Prevalent negative perception of the Croatian health care and recent Croatian accession to the EU pose a threat of losing newly graduated physicians to EU countries.

Today, health workers market is negatively affected by insufficient production due to the high cost of training, attrition, and increasing demand in the aging population. Also, an important problem is migration, which remains high on policy and research agenda ever since one of the first reports published in 1978 (1). Migration is most commonly described as negative, since the main flow of health workers is from less developed countries to more developed ones (2). For instance, in 2000 the percentage of foreign-born doctors was highest in the most developed countries, like New Zealand (46.9%), Australia (42.9%), Ireland (35.3%), Canada (35.1%), UK (33.7%), and USA (24.4%) (3). Positive aspects of migration include gaining knowledge and professional experience. They can also be seen in the remittances that emigrants send to their families left behind (4) and diaspora formation in recipient countries, which serves as a source of support and expertise transfers (5).

A considerable public concern has been raised in Croatia due to accession to the EU on July 1st 2013. Emigration waves were predicted, especially among highly educated young people, like physicians (6), although many EU countries have restricted the access to Croatian citizens by transitional arrangements until June 30, 2020 (7). At the same time, physicians’ unemployment rate in Croatia is extremely low. For example, 0.09% of the average number of all the unemployed in 2013 were doctors (8), while the unemployment among the population of medical doctors was 2.2% (9). In other EU countries similar expectations were formed upon joining the EU (10,11), but they turned out to be over-estimations, since the annual outflows from the new EU members (EU-12 countries) rarely exceeded 3% of the domestic health care workforce (12). The aim of this study was to assess the willingness and attitudes of the final year medical students from Croatia toward emigration and to compare the contemporary situation to the one a decade ago.

## Methods

This cross-sectional study was performed among the final (sixth) year medical students at all four medical schools in Croatia (Zagreb, Split, Rijeka, and Osijek). Data collection process took place in the period April-June 2013, just before graduation in July.

### Sample

Considering the uniformity of the educational and health care system in Croatia, we perceived all final-year medical students as one population and the four schools were treated as four survey sites. All students (N = 427) were invited to participate during mandatory group lectures when the survey’s purpose was explained and instructions were given. Participation in the survey was voluntary. The objective was to obtain an undirected convenience sample of at least 50% of all students. At three sites, the survey was implemented in a printed form, while in Zagreb it was implemented using the online survey platform Qualtrics (13), which was also presented during a mandatory group lecture, and students provided their answers at their own convenience.

### Questionnaire

The same self-administrated questionnaire was used in a previous study in 2003 (14), but the present study included two additional questions about students’ plans in case they did not get a specialty in the desired field in Croatia. Both questionnaires included three major sections: specialty choice, emigration preferences, and research involvement during medical studies. Students were also asked about their age, sex, grade point average, and if they failed any study year(s). Also, in 2013 survey data collection was no longer anonymous, as students were required to provide their contact details in order to investigate the outcomes of their employment seeking in the future.

The questions on emigration preferences and attitudes covered: i) the willingness to attend professional training abroad after graduation, ii) the reasons for leaving or staying in Croatia (push factors), together with the belief in getting the desired specialty in Croatia (open-ended questions), iii) the expectations in target countries (principal pull factors, open-ended question), iv) destination countries.

The majority of questions about emigration were open-ended, with an aim to capture the entire range of the opinions and attitudes, but not to influence the students’ responses in any way. All the answers to these questions were read by four co-authors (IK, MC, KB, JB). First, the authors created a list of mutually excluding categories of answers needed for the quantification of each student’s response. In the next step, three co-authors independently coded all open-ended answers according to the previously created list (MC, KB, JB). The final step was to check for the overlap between all three codes for each student (IK), and in the case of disagreement between the encoders, IK decided on the final coding.

The primary outcome variable was defined as the willingness to emigrate with the possible answers “yes,” “no,” or “not sure.” The reasons for the answer were coded according to the following categories: i) patriotism; ii) importance of relationships with family and friends; iii) higher professional demands abroad (negative connotation); iv) better professional opportunities abroad; v) better lifestyle abroad (money, standard, etc); vi) resignation with Croatian health care system; vii) uncertain and other reasons. The study was approved by the ethics board of the University of Split School of Medicine.

### Statistical analysis

Data wee summarized by survey site (school) and student subsets. The primary outcome was the prevalence of students showing willingness to emigrate permanently. It was analyzed in a multivariate logistic model in which survey site (school) was treated as a random (nesting) factor. The analysis was performed in SPSS version 17 (SPSS Inc, Chicago, IL, USA), with significance level set at *P* < 0.05.

## Results

Out of the 427 invited students, we obtained 260 valid responses (overall response rate 61%, and the lowest response of 14.5% was obtained in Rijeka). Demographic and academic characteristics are summarized in Table 1.

**Table 1:** Demographic and academic data of final year medical students in Croatia in 2013; data are median (25th-75th percentile) or count (%)

	All (N = 260)	Zagreb (N = 147)	Split (N = 55)	Rijeka (N = 11)	Osijek (N = 47)
Response rate; %	61.0	66.7	87.3	14.5	69.1
Men	81 (31.1)	41 (29.3)	20 (37.0)	4 (36.4)	16 (34.0)
Women	171 (65.8)	99 (70.1)	34 (63.0)	7 (63.6)	31 (66.0)
Age in years median	24.8 (24.4-25.3)	24.7 (24.4-25.2)	24.8 (24.3-25.5)	25.0 (24.5-25.9)	25.0 (24.6-25.6)
Grade point average	4.1 (3.8-4.4)	4.1 (3.8-4.4)	3.9 (3.6-4.1)	4.5 (4.2-4.8)	4.3 (3.9-4.5)
Ever failed a study year	57 (21.9)	36 (24.5)	14 (25.5)	0 (0.0)	7 (14.9)
Scientific involvement during studies	77 (29.6)	60 (40.8)	9 (16.4)	2 (18.2)	6 (12.8)
Most desired specialties					
Internal medicine	57 (21.9)	29 (19.7)	22 (40.0)	3 (27.3)	2 (4.3)
Surgery	30 (11.5)	19 (12.9)	2 (3.6)	2 (18.2)	7 (14.9)
Family medicine/general practice	27 (10.4)	17 (11.6)	2 (3.6)	1 (9.1)	7 (14.9)
Pediatrics	25 (9.6)	12 (8.2)	5 (9.1)	3 (27.3)	4 (8.5)
Psychiatry	15 (5.9)	14 (9.5)	1 (1.8)	0 (0.0)	0 (0.0)
Believed that they would get the desired specialty in Croatia	119 (45.8)	76 (51.7)	16 (29.1)	4 (36.4)	22 (46.8)
Willing to emigrate	90 (34.6)	55 (37.4)	17 (30.9)	3 (27.3)	15 (31.9)

The most desired specialty was internal medicine, followed by surgery and family medicine/general practice (Table 1). A total of 119 (45.8%) students believed that they would get the desired specialty in Croatia, 26 (10.0%) believed that they would not get it, while 114 (43.8%) were not certain. The main reasons for a positive response were the lack of physicians in the contemporary labor market (27.1%) and overall optimism regarding employment possibilities (26.5%). Negative responses were less frequent, with resignation due to widespread feeling of corruption and nepotism (22.4%) and overall pessimism (16.5%) as the main reasons, while 13 students expressed indecisiveness (7.6%).

A total of 90 students (34.6%) responded that they were willing to emigrate permanently (Table 1). Those who were willing to emigrate gave the following explanations: better professional opportunities (94.7%), better lifestyle (79.2%), or resignation with the Croatian health care system (90.0%) (Table 2). Students who were not willing to emigrate more frequently expressed patriotism (96.9%), importance of relationships with family and friends (100%), and the belief that working abroad demanded greater efforts and involved a greater workload than in Croatia (100%; Table 2). An additional question about the main emigration expectation, which applied only to students who claimed that they were willing to emigrate permanently, revealed principal pull reasons (70 students out of 90 responded) (Table 3). Compared to the 2003 study, the overall proportion of students willing to emigrate did not change, but the reasons for emigration and destination countries did (Table 3). Among students from 2013, the most common target countries were Germany (40%), USA and Canada (17%), and the UK (11%).

**Table 2:** Push and pull reasons associated to the willingness to emigrate, data are count (%)

Reason	Students willing to emigrate	Students unwilling to emigrate or uncertain
Patriotism	1 (3.1)	31 (96.9)
Importance of family and friends relationships	0 (0)	17 (100)
Higher professional demands in foreign country	0 (0)	11 (100)
Resignation with Croatian health care system	18 (90.0)	2 (10.0)
Ambition (better professional opportunities and development)	18 (94.7)	1 (5.3)
Better lifestyle abroad (standard of living, higher earnings...)	19 (79.2)	5 (20.8)
Uncertain and other reasons	12 (29.3)	29 (70.7)

**Table 3:** Emigration attitudes among students graduating in 2003 (14) and 2013; data are count (%)

	2003 study (N = 312)	2013 study (N = 260)
Believed that they would not get or were uncertain about getting the desired specialty in Croatia	137 (43.9)	140 (53.8)
Willing to emigrate	104 (33.3)	90 (34.6)
Principal pull factor		
Better quality of life	10 (10.5)	22 (31.4)
Better organized health care and more opportunities for professional advancement	21 (22.1)	17 (24.3)
More effort to be invested and greater professional challenge	0 (0.0)	10 (14.3)
A job in the desired field of medicine	26 (27.4)	8 (11.4)
Greater earnings	45 (47.4)	7 (10.0)
Commonest emigration targets	Slovenia (21; 20.2)	Germany (36; 40.0)
	Other EU (18; 17.3)	USA and Canada (15; 16.7)
	Non-EU countries in Europe (13; 12.5)	Other EU (13; 14.4)
	USA (10; 9.6)	UK (10; 11.1)
	Italy (6; 5.8)	Scandinavian countries (9; 10.0)

In a multivariate analysis, only two variables appeared associated with readiness to permanently emigrate: willingness to attend professional training abroad (odds ratio [OR] 6.87; 95% confidence interval [CI] 2.83-16.72) and the belief in getting the desired specialty in Croatia (OR 0.26; 95% CI 0.12-0.59; Table 4).

**Table 4:** Factors associated with willingness to emigrate, logistic regression analysis

Variable	Unadjusted odds ratio (95% confidence interval); P	Adjusted odds ratio (95% confidence interval); P
Age	0.99 (0.78-1.26); 0.933	0.80 (0.54-1.18); 0.258
Sex		
Men (Ref.)	1	1
Women	0.99 (0.57-1.74); 0.992	0.71 (0.31-1.62); 0.419
Grade point average	0.72 (0.36-1.44); 0.684	0.840 (0.27-2.60); 0.760
Ever failed a year		
No (Ref.)	1	1
Yes	0.66 (0.35-1.25); 0.202	0.50 (0.16-1.55); 0.229
Involved in research as an undergraduate student		
No (Ref.)	1	1
Yes	1.07 (0.61-1.87); 0.826	1.59 (0.67-3.77); 0.290
Interest in undertaking a temporary professional training abroad		
No (Ref.)	1	1
Yes	5.70 (3.03-10.71); <0.001	6.87 (2.83-16.72); <0.001
Belief to obtain preferred specialty in Croatia		
No (Ref.)	1	1
Yes	0.32 (0.18-0.56); <0.001	0.26 (0.12-0.59); 0.001

## Discussion

In this study, one third of the final year medical students from Croatia reported their willingness to permanently leave the country in search of employment elsewhere. Surprisingly, even though Croatia is now an EU country, the percentage of soon-to-be young doctors who considered emigration did not change in comparison to 2003 (14). This percentage is not high when compared to other European countries. For instance, as much as 62% of students from Poland estimated the likelihood of emigration to be 50% (15). Sixty percent of medical residents from Lithuania stated that they intended to emigrate, 15% of them permanently (16), as well as 45% of physicians from the Czech Republic (17). Interestingly, these forecasts often seem to overestimate the true rate of emigration, as joining the EU was rarely associated with a percentage of emigrated physicians greater than 3% (12). Similarly, one of the rare published studies on brain drain from Croatia indicated that as little as 2.8% of physicians emigrated during the 1997-2003 period, most commonly to the USA and Norway (18). Hopefully, Croatian health care, already encumbered with financial issues and a growing share of aging population (19) will not experience a major loss of graduated physicians. But since the prediction of the actual emigration extent is not possible, we believe that we will provide a more definitive answer to this question in a follow-up study in which these graduates will be re-surveyed after five years.

In comparison with previous studies among Croatian medical students, two major changes of the emigration-related patterns can be seen. First, a decade ago, students who expressed their willingness to emigrate were younger, better ranked, and those who were interested in research (20). In this study, none of these three factors played a significant role. Only two factors were associated with propensity for emigration: the wish for a temporary emigration for professional training and the belief in getting a desired specialty in Croatia (possibly a protective factor against emigration). The second major change is the choice of the target countries; in the previous study the most common target country was Slovenia (due to its proximity, language similarities, and higher salaries), followed by any of the EU countries (14). In this study, the most common target country was Germany, with as much as 40% of all answers. The most likely explanation of high interest in Germany is the long-lasting migration pattern and large Croatian community in Germany, as well as the stability of its economic situation, rarely seen in contemporary Europe.

The reasons for potential emigration among graduating medical students in Croatia were somewhat different than those reported in other European countries. For instance, major reasons for leaving Lithuania were higher salary, better professional possibilities, and better quality of life (16), similarly to the findings among Czech physicians (17) and students from Poland (15). The reasons mentioned in the previous study performed in Croatia in 2003 were better earnings (47%), getting a job (27%), better organization of the health care, and more professional opportunities (22%) (14). In contrast to these findings, contemporary students considered earnings as the least common reason for emigration, while better quality of life in the target country and better organized health care and professional development were much more common. These reasons are perhaps the reflection of the prominent resignation among students, but also in society as a whole. Moreover, medical professionals, after long and demanding studies (21), are often faced with low salaries, heavy workloads, remuneration not related to performance, and poor career prospects (22).

Numerous estimates suggest that Croatia currently seriously lacks physicians. In 2007 there was a shortage of 328 internists, 319 surgeons, 209 gynecologists, and 69 pediatricians in Croatian hospitals alone (23). A current estimate by the Chair of the Croatian Medical Chamber is that we are lacking over 4000 doctors. This shortage is also supported by the fact that there were on average only 304 unemployed doctors in 2013, compared to the average number of 345 thousand unemployed people (8).

Surprisingly, health care is among the rare labor markets in Croatia that was less affected by recession (24). This situation was also recognized by the participants in this study, 46% of whom believed that they would get the desired specialty in Croatia. But, despite the current situation in the health care labor market, contemporary students are more pessimistic than were students in 2003, when 66% of them answered that they believed they would get the desired specialty (14).

The limitations of this study include the possibility of selection bias (due to convenience sampling and low response rate in Rijeka), and information bias (student’s willingness to answer truthfully), which could have affected the results. Nevertheless, the results of this study show an interesting pattern and suggest the need for improved staff management in Croatia, which is encumbered by the lack of physicians, possibility of emigration, geographical disparities, and a dwindling and constantly reforming health care system (25).
